# Mesenchymal Stromal Cells from Osteoarthritic Synovium Are a Distinct Population Compared to Their Bone-Marrow Counterparts regarding Surface Marker Distribution and Immunomodulation of Allogeneic CD4+ T-Cell Cultures

**DOI:** 10.1155/2016/6579463

**Published:** 2016-07-19

**Authors:** Sebastien Hagmann, Claudia Rimmele, Florin Bucur, Thomas Dreher, Felix Zeifang, Babak Moradi, Tobias Gotterbarm

**Affiliations:** ^1^Clinic for Orthopedics and Trauma Surgery, Center for Orthopedics, Trauma Surgery and Spinal Cords Injury, Heidelberg University Hospital, Schlierbacher Landstrasse 200a, 69118 Heidelberg, Germany; ^2^St. Vincentius Hospital Karlsruhe, Department of Internal Medicine III, Südendstraße 32, 76137 Karlsruhe, Germany

## Abstract

*Introduction*. The participation of an inflammatory joint milieu has been described in osteoarthritis (OA) pathogenesis. Mesenchymal stromal cells (MSCs) play an important role in modulating inflammatory processes. Based on previous studies in an allogeneic T-cell coculture model, we aimed at further determining the role of synovial MSCs in OA pathogenesis.* Methods*. Bone-marrow (BM) and synovial membrane (SM) MSCs from hip joints of late stage OA patients and CD4+ T-cells from healthy donors were analysed regarding surface marker expression before and after coculture. Proliferation upon CD3/CD28 stimulation and cytokine analyses were compared between MSCs.* Results*. SM-MSCs differed from BM-MSCs in several surface markers and their osteogenic differentiation potential. Cocultures of both MSCs with CD4+ T-cells resulted in recruitment of CD45RA+ FoxP3+ regulatory T-cells. Upon stimulation, only SM-MSCs suppressed CD4+ T-cell proliferation, while both SM-MSCs and BM-MSCs modified cytokine profiles through suppressing IL-2 and TNF-*α* as well as increasing IL-6 secretion.* Conclusions*. Synovial MSCs from OA joints are a unique fraction that can be distinguished from their bone-marrow derived counterparts. Their unique ability to suppress CD3/CD28 induced CD4+ T-cell proliferation makes them a potential target for future therapeutic approaches.

## 1. Introduction

While osteoarthritis remains one of the most frequent musculoskeletal diseases [1], accounts for a vast number of hospitalization admissions, and is a frequent cause of disability [2], it basically remains an incurable disease. While total joint replacement has provided more than satisfactory results for some joints, it remains an end-stage procedure. Moreover, the rising number of joint replacements will cause an important increase in revision surgery in the future [3], with all its medical and socioeconomic impacts. While currently available drugs and activity may provide temporary relief and certain factors may slow down the progression of the disease, the cascade of joint destruction cannot be detained.

In the search for new strategies of disease modification, recently, attention has turned towards the fact that inflammation and synovitis especially may be more important in the progression of osteoarthritis than initially estimated [4, 5]. While synovial inflammation in osteoarthritis has already been reported more than 20 years ago [6, 7] and since then several other studies have underlined the importance of inflammatory infiltrates in the disease [8–10], no significant advances in therapeutical approaches have been made in this regard. Only recently synovitis has been shown to promote cartilage degeneration in osteoarthritis [11]. However, the pathophysiology of synovitis in OA, in contrast to rheumatoid arthritis (RA), is only marginally understood. While mesenchymal stem or stromal cells (MSCs), for instance, have been identified as potential key players in the immunomodulation of RA [12, 13], their role in the inflammatory milieu of the osteoarthritic joint is mostly unknown.

We have recently reported that MSCs derived from the synovium and bone marrow of osteoarthritis patients may be candidates for immunoregulation in the affected joint, based on our findings that these cells effectively regulate regulatory T-cells (Tregs) in an in vitro coculture model [14]. Recent findings of our group also suggest that, comparable to RA, OA joints show an accumulation of Tregs and, to a lesser extent, of CD4+ T-cells in the synovial membrane [15]. The significance of these findings for possible therapeutic approaches however is yet unclear; a basic understanding of how MSCs and T-cells may interact in osteoarthritis therefore is imperative.

The aim of this study was to determine the effect of MSCs derived from the synovium and bone marrow of osteoarthritis patients on CD4+ T-cells in an allogeneic coculture model. We focussed on a regulatory T-cell subset analysis, changes in cytokine secretion and proliferation upon stimulation.

We here show for the first time that MSCs derived from osteoarthritic synovium are distinct from their bone-marrow counterparts with regard to their surface marker expression, the ability to suppress CD3/CD28 triggered CD4+ T-cell proliferation, and IL-6 secretion in CD4+ cocultures. Our data suggests that these cells could be potential targets for cell-based immunomodulatory approaches.

## 2. Methods

### 2.1. Patients and Blood Donors

MSCs were isolated from bone marrow and synovial membrane of 10 patients (mean age: 65.8 years; 5 females and 5 males). Both tissues were collected from the same patients during total hip arthroplasty for primary late-stage osteoarthritis (radiologic grades III and IV according to the Kellgren/Lawrence score).

Lymphocytes were extracted from whole blood samples of healthy donors (26.8 years; 9 females and 2 males). Exclusion criteria for OA patients were a history of acute or chronic infections, cancer, and rheumatic diseases. In addition to these criteria, exclusion criteria for the blood donors included a history of cartilage injury and osteoarthritis as well.

### 2.2. Ethics, Consent, and Permissions

All patients and blood donors provided informed consent. All procedures were in accordance with the Helsinki Declaration of 1975, as of its latest revision. The study protocol was approved by the Ethics Committee of the University of Heidelberg, Germany (S113-2009).

### 2.3. Mesenchymal Stromal Cells (MSCs)

During preparation of the femur, bone marrow was collected into heparinised isotonic saline solution. The synovial membrane was dissected from joint capsule resections under sterile conditions. A part of the synovium was stored overnight in DMEM-LG and digested with collagenase B (Sigma Aldrich, St. Louis, MO, USA) and hyaluronidase (Roche, Basel, Switzerland) in DMEM-LG (Dulbecco's Modified Eagle's Medium low glucose, Life Technologies, Carlsbad, CA, USA) for two hours the following day before being used for flow cytometry analysis (see below). The rest of the synovium was digested overnight as described above. For the isolation of MSCs, the mononuclear cells (MNCs) fraction of both bone-marrow (BM) and synovium (SM) derived cells was isolated by Ficoll Paque Plus gradient centrifugation (GE Healthcare, Chalfont St Giles, Great Britain). BM-MNCs and SM-MNCs were suspended at a density of 1.25 × 10^5^ cells into Cellstar T75 cell culture flasks (Greiner Bio-One, Kremsmünster, Austria) that had been previously coated with 5 mL of a 0.1% gelatine (Sigma Aldrich, St. Louis, MO, USA) solution diluted in phosphate buffered saline (PBS, Life Technologies, Carlsbad, CA, USA). The MSC expansion medium consisted of a variation of Embryonal Stem Cell Expansion (ES) medium, containing 500 mL DMEM 4.5 g/L (high) glucose without L-Glutamine (Life Technologies, Carlsbad, CA, USA), 75 mL FCS (Life Technologies, Carlsbad, CA, USA), 6 mL penicillin/streptomycin (P/S, Biochrom, Berlin, Germany), 6 mL L-Glutamine (Life Technologies, Carlsbad, CA, USA), 6 mL nonessential amino acids (NEAA, Life Technologies, Carlsbad, CA, USA), 600 *μ*L 2-Mercaptoethanol (Life Technologies, Carlsbad, CA, USA), and 20 *μ*L/50 mL FGF basic (FGF-2, Acris, Herford, Germany). After 24 hours, medium was replaced and nonadherent cells were discarded. Medium replacement was carried out every two to three days afterwards. At 80% confluence, the medium was collected, and the cells were washed with PBS (Life Technologies, Carlsbad, CA, USA). MSCs were then detached with trypsin 0.5% (Biochrom, Berlin, Germany), washed with complete medium, and counted after staining with trypan blue 0.4% (Sigma Aldrich, St. Louis, MO, USA). MSCs were then replated at a density of 5 × 10^3^/cm^2^ in gelatinated cell culture flasks and cultured until the end of passage 1, after which all MSCs were frozen in DMSO (Sigma Aldrich, St. Louis, MO, USA) in order to coordinate that SM-MSCs and BM-MSCs could be used simultaneously. After thawing and expansion to P2, some of the cells were used for flow cytometry (see below) and coculture assays (see below) while another part (*n* = 5 donors) underwent osteogenic, chondrogenic, and adipogenic differentiation assays according to standard protocols previously described [16].

### 2.4. CD4+ T-Cell Isolation

Whole blood cell samples were treated by Ficoll Paque Plus gradient centrifugation (GE Healthcare, Chalfont St Giles, Great Britain). The MNC fraction was collected and washed in PBS (Life Technologies, Carlsbad, CA, USA). CD4+ cells were then isolated from the MNCs using the CD4 Isolation Kit II (Miltenyi Biotec, Bergisch Gladbach, Germany) according to the manufacturer's protocol. In brief, after 10 min of incubation with the Biotin antibody cocktail, the cells were incubated for another 15 min with anti-biotin microbeads. The cells were then washed and resuspended in the MACS separation buffer (Miltenyi Biotec, Bergisch Gladbach, Germany). Magnetic negative selection isolation for CD4+ T-cells was performed using LS columns and a MidiMACS*™* separator (all from Miltenyi Biotec, Bergisch Gladbach, Germany).

### 2.5. MSC/CD4+ Cocultures

All coculture assays were conducted in triplicate. For these assays, the cells (MSCs, T-cells, and cocultures) were cultured in DMEM-LG (Life Technologies, Carlsbad, CA, USA) with 55 mL FCS (Life Technologies, Carlsbad, CA, USA) and 5,5 mL penicillin/streptomycin (Biochrom, Berlin, Germany) per 500 mL DMEM-LG for 5 days. The experiments were carried out in 24-well plates (Nunclon, Sigma Aldrich, St. Louis, MO, USA) with 75,000 MSCs and 150,000 CD4+ T-cells per well, in a total of 1500 *μ*L of medium. MSC monocultures (75,000 cells per well) and CD4+ T-cell monocultures (150,000 cells per well) were used as controls. Medium replacement was conducted at d2. Therefore, the assays were centrifuged at 1300 rpm for 8 min, and 1350 *μ*L of the supernatant was carefully collected, frozen, and replaced. The same procedure was performed at d5. Afterwards, however, the nonadherent cells were collected and underwent flow cytometry (see below). The adherent cells were detached as described above, washed, and analyzed by flow cytometry (see below).

### 2.6. Proliferation Assays and T-Cell Activation

Lymphocyte proliferation was detected by carboxyfluorescein diacetate succinimidyl ester (CFDA SE) staining with the Vybrant® CFDA SE Cell Tracer Kit (Life Technologies, Carlsbad, CA, USA) with a variation of manufacturer's instructions. In brief, after the CD4+ lymphocytes were washed and counted, they were resuspended in PBS at a density of 1 × 10^6^/mL. Bovine serum albumin (BSA, Sigma Aldrich, St. Louis, MO, USA) was then added to obtain a concentration of 0.1%. 18 *μ*L of DMSO was added to 500 *μ*g of the CFDA SE probe, and 0.1 *μ*L of the stock solution was added per 1 million cells. The cells were incubated at 4°C for 5 min; then incubation was stopped by adding a buffer consisting of 135 mL PBS and 15 mL FCS. The cells were then washed thrice, resuspended in whole medium, and counted. Lymphocytes thus treated were then activated (see below) and used in coculture assays or as controls (*n* = 5 per group, all assays conducted in triplicate).

T-cell activation was performed after CFDA SE staining with the Dynabeads® Human T Activator CD3/CD28 kit (Life Technologies, Carlsbad, CA, USA) according to manufacturer's instructions. Nonactivated cells were used as controls.

Analysis of proliferation was performed by defining a cutoff on unstimulated CD4+ lymphocytes at d5 where 1% of the cells were considered positive (99% positive cells right from the gate, see below), as described in [17].

### 2.7. Flow Cytometry

Before flow cytometry analysis, all cells were washed with PBS and resuspended in autoMACS® running buffer (Miltenyi Biotec, Bergisch Gladbach, Germany). The antibodies and isotype antibodies used for flow cytometry are listed in Supplemental Table 1 in Supplementary Material available online at http://dx.doi.org/10.1155/2016/6579463. Dead/live staining was conducted with a 7-amino-actinomycin D (7-AAD) Viability Staining Solution (eBioscience, San Diego, CA, USA). For the assessment of all cells, FcR block was performed by incubation with FcR blocking reagent (Miltenyi Biotec, Bergisch Gladbach, Germany) for 8 min. Multicolour flow cytometry was conducted on a MACSQuant*™* analyser (Miltenyi Biotec, Bergisch Glattbach, Germany). FoxP3 intracellular staining was performed after fixation using the FoxP3 staining buffer set (Miltenyi Biotec, Bergisch Glattbach, Germany) according to the manufacturer's protocol. The MACSQuantify 2.1 software (Miltenyi Biotec, Bergisch Glattbach, Germany) was used for data analysis. Positive fluorescence was defined as any event above the background fluorescence in a histogram. Background fluorescence was defined by a cutoff where 99.5% of the background fluorescence events matched to isotype antibody results were marked negative.

### 2.8. Cytokine Analysis

Cytokine detection in the culture supernatants for IL-2, IL-4, IL-6, IL-10, IL-17a, TNF-*α*, and IFN-*γ* was simultaneously conducted with the human TH1/TH2/TH17 Cytokine and a separate TGF-*β* kit (BD Biosciences, Heidelberg, Germany), using a MACSQuant analyser and the MACSQuantify 2.1 software (Miltenyi Biotec, Bergisch Glattbach, Germany) according to the manufacturer's protocol. For data analysis, the FCAP Array Software, Version 1.0.1 (BD Biosciences, Heidelberg, Germany), was used. The assays were performed with undiluted supernatants or supernatants diluted to 1 : 10 or 1 : 100 with PBS (Invitrogen, Karlsruhe, Germany) until matching the standards.

### 2.9. Statistical Analysis

Of all triplicate assays, means were calculated. All data were at first tested upon normal distribution by a graphic display (QQ-plot, histogram, or box plot), a ratio analysis, and Kolmogorov-Smirnov (with Lilliefors significance correction) as well as Shapiro-Wilks testing. As all data sets contained paired data, paired samples tests were employed. For nonparametric data, Wilcoxon signed ranks test for comparison of two groups and Friedman tests for comparison of multiple groups were performed (MSC surface markers). *p* values of <0.05 were considered significant.

For parametric data Student's *t*-test for comparison of two groups and analyses of variance (ANOVA) for multiple groups were performed (MSC osteogenesis, cytokine levels, lymphocyte surface markers, and quantitative CFSE analysis). *p* values of <0.05 were considered significant for *t*-tests and ANOVA group-to-group comparisons (after Bonferroni correction). All calculations were performed using the SPSS software (SPSS Inc., released 2009, PASW Statistics for Windows, Version 18.0, Chicago).

## 3. Results

### 3.1. Flow Cytometric Analysis of the MNC Fractions in Synovial Membrane

**Figure 1:**
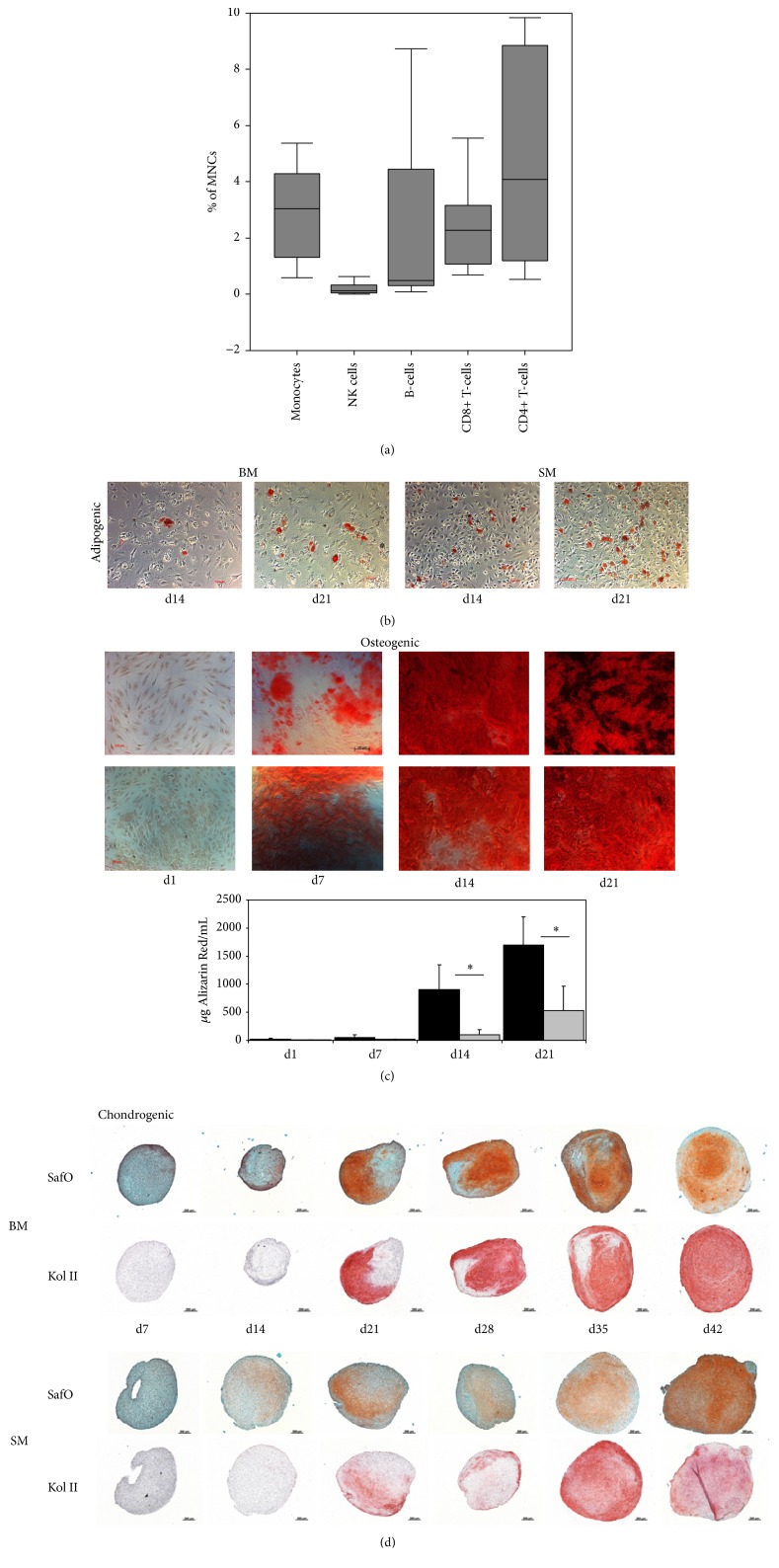
Mononuclear cell infiltration in the synovial membrane and MSC differentiation results. (a) The boxplot diagram displays the percentages of positive cells for the mononuclear cell fraction, CD14+ monocytes, CD16+CD56+ NK cells, CD4+ and CD8+ T-cells, and B-cells in the synovial membrane of 10 patients enclosed in the study. (b–d) Differentiation into the three lineages was successful in all MSCs (*n* = 5 patients per assay), while important donor-dependent variations were observed. (b) The figure shows representative results of adipogenic differentiation at d14 and d21 as defined by Oil Red O-stained lipid vacuoles. (c) Osteogenic differentiation was determined by calcium deposition through Alizarin Red staining (quantitative analysis of the reextracted dye is depicted in the diagram) and was enhanced in BM-MSCs compared to SM-MSCs. (d) Chondrogenic differentiation was assessed by Safranin O and Collagen II staining. *∗* refers to significant differences between the groups (*p* < 0.05).

In order to assess the inflammatory activity inside the joint at the time of surgery, a part of the digested synovial membrane underwent flow cytometric analysis for the percentages of mononuclear cells, CD14+ monocytes, CD16+CD56+ NK cells, CD4+ and CD8+ T-cells, and B-cells. While, with the exception of one patient, the percentage of mononuclear cells showed only moderate variations in the synovial membrane (85.2 ± 19.7% of total cells, *n* = 10), more important variations were observed for the respective subpopulations mentioned above (*n* = 10, Figure 1(a)).

### 3.2. Differences in Naïve MSC Differentiation Results and Surface Marker Distribution

All MSCs showed the minimal criteria for MSCs as defined by the International Society for Cellular Therapy (ISCT) [18]. No difference in plastic adherence was observed between SM-MSCs and BM-MSCs. Chondrogenic, osteogenic, and adipogenic differentiation were highly donor-dependent; however, the three were successful in both cell types (Figures 1(b)–1(d), *n* = 5 for all differentiation assays). Quantitative analyses of Alizarin Red contents showed a higher mean osteogenic differentiation of BM-MSCs compared to SM-MSCs (Figure 1(c), *p* = 0.022 for d14 and *p* = 0.019 for d21).

**Figure 2:**
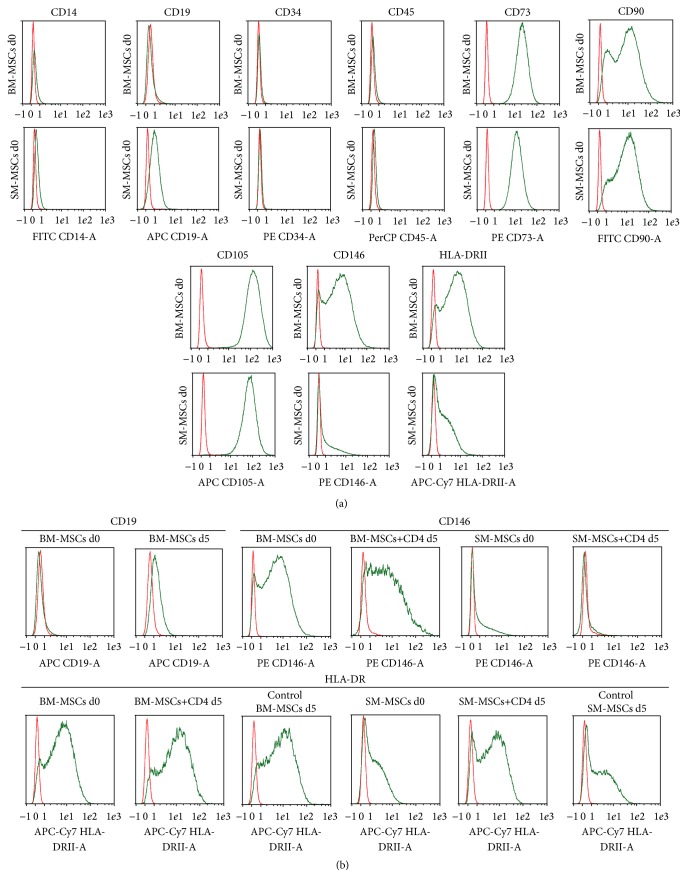
Surface marker expression on SM-MSCs and BM-MSCs. (a) Representative histograms of surface marker expression on SM-MSCs and BM-MSCs as detected by flow cytometry before coculture or monoculture. Red: background fluorescence; green: surface marker. (b) Marked differences between the groups were observed for CD19, CD146, and HLA-DR, as shown by representative histograms. See Table 1 for means and standard deviation.

The mean percentages of positive cells for the tested MSC surface markers in naïve BM-MSCs and SM-MSCs at d0 are displayed in Table 1 (*n* = 10), and representative histograms are displayed in Figure 2(a). While the phenotypic prerequisites for MSCs as defined by the ISCT were present on both naïve SM-MSCs and BM-MSCs, significant differences between the two were detected for CD90, CD146, and HLA-DR (Table 1, Figure 2(a)), with SM-MSCs showing a significantly higher CD90 expression (+3.9% positive cells; *p* = 0.022) and a significantly lower HLA-DRII (−21.72% positive cells; *p* = 0.007) and CD146 expression (−63.46% positive cells; *p* = 0.005). These three surface markers, together with CD19, were also the ones that showed the greatest variability between the donors, with HLA-DR clearly leading (ranging from 9.11% HLA-DR positive cells in the synovial membrane of one patient to 92.46% in bone marrow of another patient). Also, the number of cells in late apoptosis or necrosis, as defined by 7-AAD staining, was significantly lower in SM-MSCs.

### 3.3. Differences in MSC Surface Marker Expression before and after Lymphocyte Coculture

MSCs in late apoptosis or necrosis, as reflected by 7-AAD staining, were significantly lower in the CD4+ coculture groups compared to control MSCs at d5 (Table 1). Important differences in the expression of several MSC surface markers could be observed between d0 and d5 (Table 1, Figure 2(b)) and between control MSCs and MSCs cocultured with CD4+ T-cells.

While subtle yet significant differences between the surface marker expressions of CD34, CD73, CD90, and CD105 could be observed between d0 and d5 in cocultures (Table 1), we observed more important changes in the expression of other surface markers, namely, a significant decrease of CD14 expression in both cocultured and control BM-MSCs and SM-MSCs (Table 1), while only BM-MSCs showed an increased CD19 expression at d5 in monoculture and coculture (Table 1, Figure 2(b)). The most important differences were observed for CD146, with a drastic decrease in surface expression upon coculture with MSCs from both bone marrow and synovium (Table 1, Figure 2(b)) when compared to control MSCs and expression levels at d5, and HLA-DR. The latter showed a significant increase in surface marker expression in MSCs cocultured with CD4+ T-cells (Table 1, Figure 2(b)).

### 3.4. Lymphocyte Markers

The mean percentage of CD4+ cells before the cocultures was 95.17 ± 2.37%. No significant differences regarding the CD4+ percentages were observed between T-lymphocytes at d0 and d5 and the coculture groups (*p* = 0.384–*p* = 1.00, *n* = 10).

**Figure 3:**
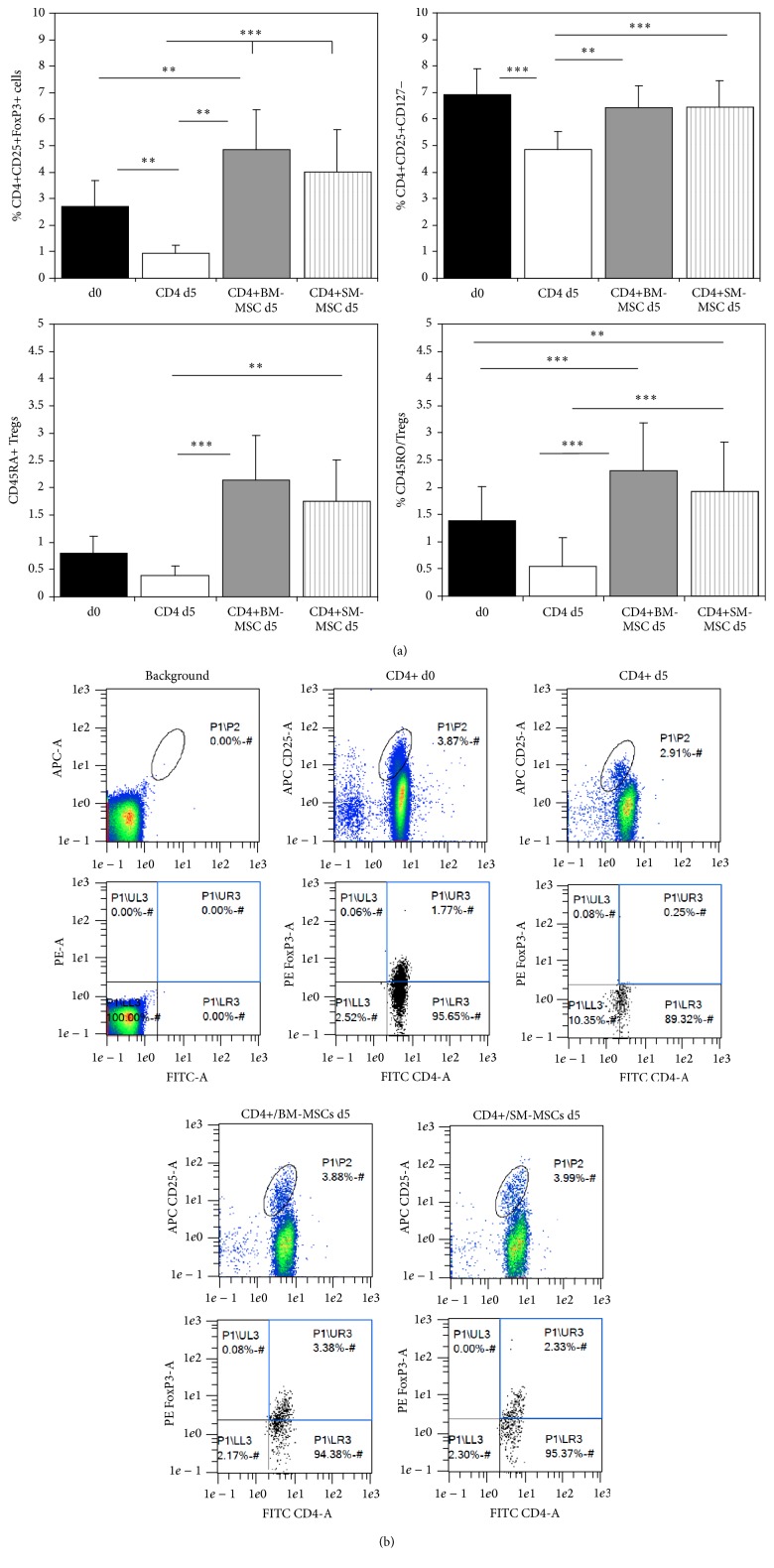
Surface marker expression on CD4+ T-cells in monocultures and MSC-cocultures. (a) The diagrams display the mean positive cells for CD4+CD25+FoxP3+, CD4+CD25+CD127-, CD45R0+ Tregs, and CD45RA+ Tregs as detected by flow cytometry (*n* = 10). ^*∗∗*^ *p* < 0.01 and ^*∗∗∗*^ *p* < 0.001. (b) Representative flow cytometry plots for background fluorescence, CD4+ T-cell monocultures at day 0 and day 5, and the respective BM-MSC and SM-MSC cocultures at day 5. Lymphocytes were gated for CD4, CD25, and FoxP3.

Coculture of CD4+ T-cells with OA-derived BM-MSCs led to a significant elevation in the proportion of CD4+CD25+FoxP3+ T-cells (Figures 3(a) and 3(b), *p* = 0.0015) compared to the initial percentage at d0. There was a tendency towards the same effect in SM-MSC cocultures (*p* = 0.1). CD4+ T-cell monocultures showed a significant decrease in the CD4+CD25+FoxP3+ T-cell percentages compared to d0 (*p* = 0.013). Compared to control CD4+ monocultures, the percentages of FoxP3 Tregs in both SM-MSC and BM-MSC cocultures were significantly higher (both *p* < 0.001).

Compared to control CD4+ monocultures, the addition of MSCs resulted in a significant increase in CD45R0+ and CD45RA+ Tregs (CD4+CD25 high) at day 5 (Figure 3(a)). Additionally, an increase in CD45RA+ Tregs upon addition of MSCs could be observed compared to the initial d0 values (*p* < 0.001 for BM-MSCs and *p* = 0.0041 for SM-MSCs), which was not the case for CD45R0+ Tregs (*p* = 0.053 for BM-MSCs and *p* = 0.72 for SM-MSCs).

### 3.5. Stimulated Lymphocyte Cultures

**Figure 4:**
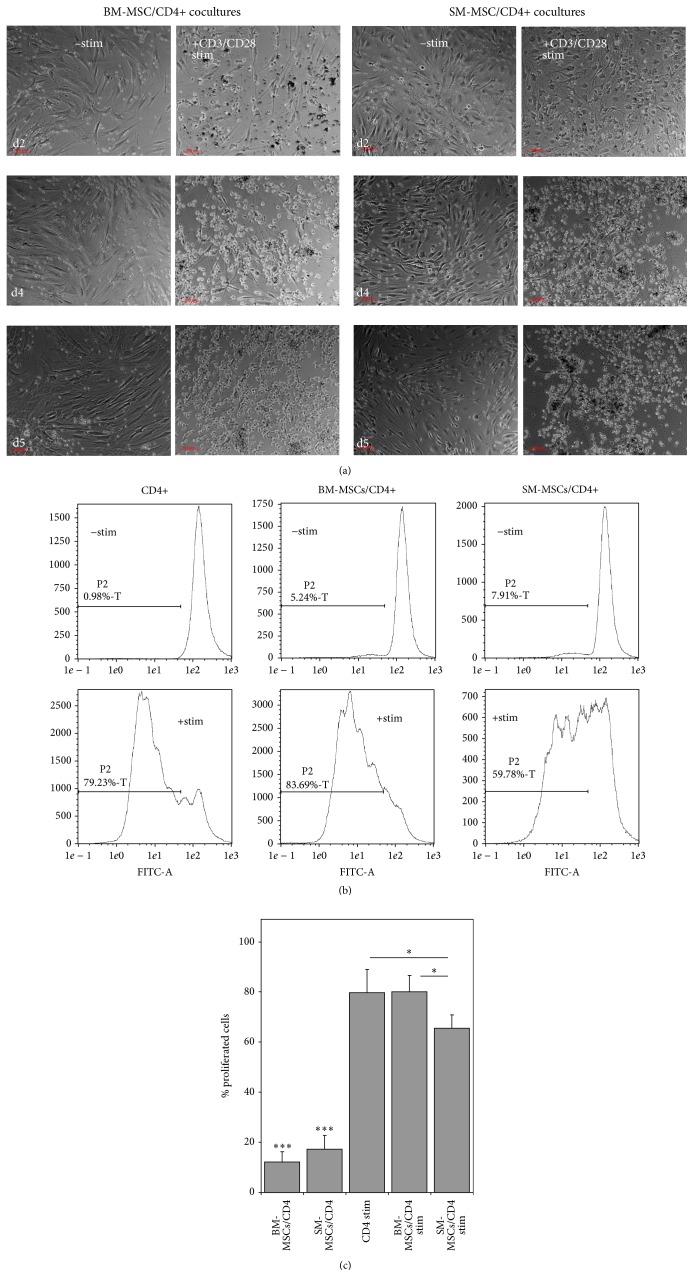
Evaluation of MSC/CD4+ T-cell interaction upon T-cell stimulation. (a) Representative photographs at d2, d4, and d5 of coculture for bone-marrow (BM) and synovial membrane (SM) derived MSCs are displayed. Index: 100 *μ*m. (b) Representative histograms from one CFSE assay (total triplicate assays *n* = 5) for unstimulated CD4+ T-cells, unstimulated CD4+BM-MSC and SM-MSC cocultures, and the respective CD3/CD28 stimulated cultures. (c) The histogram displays the mean percentage of proliferated cells in five triplicate CFSE assays (see Methods for gating). ^*∗*^ *p* < 0.05 and ^*∗∗∗*^ *p* < 0.001.

Upon CD3/CD28 stimulation, CD4+ T-cells started to proliferate in monocultures as well as in the cocultures (*n* = 5 per group, all assays conducted in triplicate). Proliferation was clearly visible in light microscopy and lymphocytes tended to coat the MSCs in coculture (Figure 4(a)). Compared to unstimulated CD4+ monocultures, both BM-MSCs and SM-MSCs cocultures showed a moderate increase in lymphocyte proliferation (Figure 4(b)). Upon CD3/CD28 stimulation, a significant increase in proliferation could be observed (*p* < 0.001 for all comparisons of unstimulated versus stimulated groups). While stimulated CD4+ monocultures and BM-MSC cocultures showed a comparable increase in proliferation (*p* = 0.416), synovium derived MSCs suppressed CD4+ T-cell proliferation compared to both CD4+ monocultures and BM-MSC cocultures (Figures 4(b) and 4(c), *p* = 0.023 and *p* = 0.018, resp.).

### 3.6. Cytokine Profiles

In supernatants of BM-MSC and SM-MSC monocultures, IL-2, IL-4, IL-17a, TNF-*α*, and IFN-*γ* levels were below the theoretical detection limit of the CBA kit. Only relevant amounts of IL-6 and TGF-*β* could be detected in these cultures. Statistical differences between IL-6 and TGF-*β* levels of CD4+ T-cells and MSC monocultures could not be detected, and treatment of MSC monocultures with the stimulation reagents did not result in differences in cytokine levels compared to control MSCs (data not shown).

**Figure 5:**
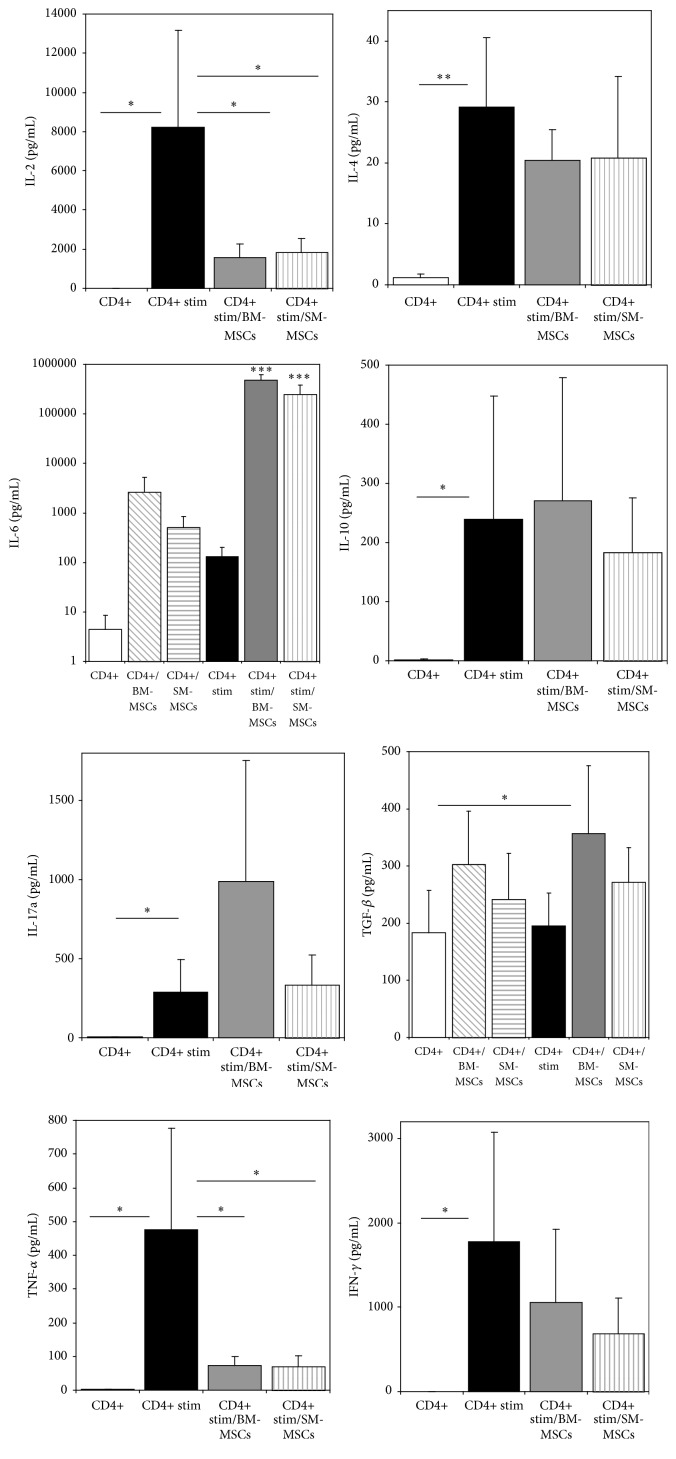
Cytokine levels in CD3/CD28- stimulated and control CD4+ T-cells and the respective MSC cocultures. CD3/CD28 stimulation resulted in an increase of IL-2, IL-4, IL-6, IL-10, IL-17a, TNF-*α*, and IFN-*γ* secretion. While for IL-2 and TNF-*α* cocultivation with MSCs resulted in a significant decrease in CD4+ T-cell cytokine secretion, a significant increase in IL-6 secretion in unstimulated and stimulated cocultures could be observed. Due to the high IL-6 levels in the stimulated cocultures, this diagram is displayed with a logarithmic *y*-scale. ^*∗*^ *p* < 0.05, ^*∗∗*^ *p* < 0.01, ^*∗∗∗*^ *p* < 0.001.

The mean IL-2 levels of unstimulated CD4+ T-cell as well as CD4+/MSC cocultures were low, with only some of the patient's MSCs showing secretion above the theoretical detection limit. While unstimulated cultures showed IL-4, IL-10, IL-17a, TNF-*α*, and IFN-*γ* levels beyond the theoretical detection limit, significant increases of these cytokines were observed upon stimulation in the CD4+ monocultures (Supplemental Table 2, Figure 5; IL-2: factor 3832.4, *p* = 0.021; IL-4: factor 23.7, *p* = 0.005; IL-10: factor 101.2, *p* = 0.043; TNF-*α*: factor 278.3, *p* = 0.024; IL-17a: factor 90.9, *p* = 0.03; IFN-*γ*: factor 2438.8, *p* = 0.024). No significant differences regarding IL-6 secretion could be detected between stimulated and unstimulated CD4+ cells and the respective MSC cocultures (Figure 5, Supplemental Table 2, all *p* = 1.0).

Due to high variations in cytokine secretion among the cultures, significant differences upon MSC addition to stimulated CD4+ cultures could only be detected for IL-2, IL-6, and TNF-*α* (Figure 5). Cocultivation with MSCs resulted in a decrease in IL-2 production of factor 5.4 in BM-MSC and factor 4.1 in SM-MSCs (Figure 5, *p* = 0.011 and *p* = 0.014 compared to CD4+ monocultures) and in a decrease of TNF-*α* secretion of factors 6.38 (*p* = 0.01) and 6.26 (*p* = 0.01), respectively.

In contrast, stimulated CD4+ cultures with MSCs showed an important increase in IL-6 production compared to unstimulated cultures and stimulated CD4+ T-cells (*p* < 0.001 for all groups). The amount of IL-6 secreted by BM-MSCs in the stimulated CD4+ coculture was significantly higher than in the respective SM-MSC cocultures (Figure 5, *p* = 0.0017). No differences in TGF-*β* secretion upon stimulation were observed for CD4+ T-cells, while in BM-MSC/CD4+ cocultures elevated levels of this cytokine could be detected (*p* = 0.048 compared to CD4+ cells, *p* = 0.079 compared to stimulated CD4+ T-cells).

## 4. Discussion

Not only is osteoarthritis the most common joint disease worldwide, but also its treatment devours enormous medical and socioeconomic resources. Late-stage osteoarthritis can only be efficiently treated by arthroplasty, which is often referred to as the “death of the joint.” Although effective in most cases, treatments beyond arthroplasty, especially for early-stage osteoarthritis, need to be developed. Cell-based approaches involving mesenchymal stromal cells have been proposed mainly in the context of tissue regeneration; however, given their immunomodulatory potential, understanding the interactions of immune cells and MSCs may pave the way for novel therapeutic approaches as well.

We believe that we are the first ones to report that MSCs from osteoarthritic synovium are a distinct population compared to bone-marrow derived cells from the same patients with regard to their immunomodulatory properties and surface marker profiles. We have previously reported that MSCs derived from OA patients maintain a regulatory phenotype in Tregs [14]. In the study presented here, we focussed on differences between MSCs derived from the joint and bone marrow, analysing surface markers and differentiation as well as giving more detailed insights into the communication between T-cells and MSCs.

SM-MSCs clearly showed higher CD90 and lower CD146 and HLA-DR expression than BM-MSCs. In the case of CD90, it seems that SM-MSCs are simply a more homogeneous population, while CD146 expression can be seen as a true indicator of SM-MSCs being a distinct subpopulation compared to BM-MSCs. While different studies report the occurrence of CD146 expression on human MSCs between 15% and percentages comparable to those observed in our BM-MSCs [19, 20], this marker is also highly donor-dependent and depends on FGF-2 administration to the medium [16]. The culture conditions for all MSCs being the same, only the origin of the cells can thus be held accountable for the differences observed. CD146 expression has been associated with higher potency in BM-MSCs [21], which could be responsible for the reduced osteogenic potential of SM-MSCs in our study.

The differentiation into the adipogenic, osteogenic, and chondrogenic lineage mainly proves, along with the presence and absence of certain surface markers, that both BM-MSCs and SM-MSCs fulfil the criteria proposed by the ISCT [18]. An exception to matching these criteria is the presence of HLA-DRII on the cells used in our experiments.

We were able to show that HLA-DR (MHCII) expression on SM-MSCs is consistently lower than in BM-MSCs from the same patients. However, HLA-DR expression was relatively high on both BM-MSCs and SM-MSCs. According to the ISCT criteria, MSCs must be negative for HLA-DR to qualify as such [18]. It has, however, been shown that HLA-DR expression is not negative, but low in most MSC populations, and is highly variable in murine, equine, and human MSCs depending on inflammatory stimuli as well as several cytokines, such as IFN-*γ*, TGF-*β*, and FGF-2 [22–24], the latter being part of our culture medium. Jo et al. described that freshly isolated SM-MSCs express numerous surface markers that are considered “negative” markers, such as CD14, CD34, CD45, and HLA-DR [25]. In contrast to our experiments, however, HLA-DR expression disappeared after the first passage. The fact that an increase in HLA-DR expression was observed in control MSC monocultures as well could be explained by upregulation upon changes in cell density in the coculture assays [23]. It was however not our intention to derive the influence of these factors to HLA expression, but to compare SM-MSCs and BM-MSCs from the same patients. For these cells, the exact same culture conditions were applied. Additionally, it has been shown that MSCs that have been conditioned to show full MHCII expression still escape allorecognition [26] and thus may be useful for therapeutic approaches. Nonetheless, the fact that HLA-DR expression is culture dependent should not be neglected during considerations of using MSCs in patients, especially in allogeneic strategies. Choosing an allogeneic approach has practical reasons in the first place: given the fact that THA is associated with substantial blood loss, the amount of additional blood to collect sufficient samples of T-cells causes ethical problems. Higher volumes of blood can be obtained in healthy subjects, which is in favor of an allogeneic approach. Also, we do not know whether osteoarthritis itself may affect lymphocyte function, which is why we chose to investigate the effects on lymphocytes from healthy donors. Allogeneic cultures have been shown to be adequate models for T-cells/MSC interactions in a number of studies [27–29].

Coculturing MSCs with CD4+ T-cells led to a significant reduction of T-cells in late apoptosis and necrosis, as defined by 7-AAD staining, indicating that MSCs stabilize and protect CD4+T-cell populations in vitro, which is consistent with the findings of another group [29]. Interestingly, in all cultures involving synovium derived MSCs, a reduction of cells in late apoptosis and necrosis could be observed compared to BM-MSCs. This finding has not been reported so far and adds to the distinct properties of SM-MSCs exerted in our study. The underlying mechanism however remains unexplained, given the fact that the only cytokines detected in unstimulated monocultures and CD4+ cocultures, IL-2, IL-6, and TGF-*β*, showed no differences between SM-MSCs and BM-MSCs.

In our study, BM-MSCs induced CD4+CD25+FoxP3+ regulatory T-cells compared to the percentages at d0, while both SM-MSCs and BM-MSCs prevented the drastic decrease of this population observed in CD4+ monocultures. While we believe that we are the first ones to report on this effect in the context of osteoarthritis, these results match with findings from a number of studies that showed that MSCs could induce FoxP3+ Tregs from the CD4+ fraction [30–32]. We believe it is an important finding that the recruiting of activated T-cells would not have been documented if we had only looked at CD127- cells, which is reported to be inversely correlated with FoxP3 expression [28]. This indicates that FoxP3 must be considered an essential marker for Treg activation.

Compared to the initial percentages of CD45R0+ (memory) Tregs at d0, both SM-MSCs and BM-MSCs retained this population in cocultures. A significant increase in the CD45RA+ fraction could be observed upon coculture with MSCs, suggesting that the observed increase in FoxP3+ Tregs was due to an increase of naïve regulatory T-cells. This effect had been demonstrated by Di Ianni et al. when coculturing BM-MSCs with CD3+ T-cells [28]. We believe that we are the first ones to confirm this effect in the context of osteoarthritis. Animal studies will of course have to prove whether these in vitro findings can be applied to the complexity of the inflamed osteoarthritic joint; however, we believe that our results show that synovial MSCs may play an important role in modulating the inflammatory processes taking place in the joint.

In this context, we here report for the first time that MSCs derived from the synovium of osteoarthritic joints are able to suppress CD4+ T-cell proliferation upon stimulation whereas bone-marrow derived MSCs fail to do so. Interestingly, this effect was shown even after the cells had been expanded in vitro to the end of passage 2. This is an important finding suggesting that SM-MSCs can be safely isolated and expanded and still exert important immunomodulatory properties, which is an important aspect when considering MSC-based therapeutic approaches.

While an important study conducted by Yoo et al. was able to show that MSCs from adipose tissue, umbilical cord blood, and Wharton's jelly showed immunomodulatory properties comparable to bone-marrow derived MSCs [33], this study did not include synovium derived MSCs. Synovium derived MSCs from patients with rheumatoid arthritis (RA) have been shown to suppress T-cell proliferation in vitro comparable to SM-MSCs from healthy donors [34]. However, no comparable literature exists for patients with OA. BM-MSCs have been shown to be able to suppress T-cell proliferation in vitro in other studies [35]. It is therefore the question whether methodological differences or the disease conditions are to account for the inability of BM-MSCs to suppress T-cell proliferation in our experiments. The fact that SM-MSCs were able to suppress T-cell proliferation in vitro compared to monocultures of CD4+ cells may be a result of having been exposed to the inflammatory joint milieu. This seems to apply even given the fact that the cellular composition of mononuclear infiltration was highly variable among the patients. An important result of our experiment is therefore that although varying degrees of inflammation in the joints can be observed, SM-MSCs consistently suppress CD3/CD28 induced proliferation after in vitro expansion. Future experiments will have to determine whether some sort of “imprinting” is the central mechanism behind the differences observed between SM-MSCs and BM-MSCs. This however would involve comparing MSCs from healthy synovium, which is difficult to realize due to ethical considerations. Given the fact that in up to 65% of all arthroscopic procedures cartilage lesions can be detected, this collective can hardly be considered as healthy controls [36].

Finally, our study suggests that, while without stimulation, an important part of the interaction of MSCs and T-cells relies on TGF-*β* and, more importantly, IL-6. Stimulation of CD4+ T-cells with CD3/CD28 leads to an important increase in the secretion of cytokines of both pro- and anti-inflammatory nature. While MSCs did not alter the secretion of IL-4, IL-10, IL-17a, and IFN-*γ* in stimulated CD4+ cocultures, they led to a significant decrease in IL-2 and TNF-*α* secretion, while IL-6 production was drastically increased.

The suppression of TNF-*α* secretion by stimulated T-cells can be an indicator of an important role of MSCs in protecting the joint from the TNF-*α*-mediated production of catabolic proteases by chondrocytes [37]. Synovitis generally is associated with disease progression through cartilage loss in OA [11, 38], and cytokine-dependent degeneration may be of importance in this regard. In a phase I study, Jo et al. were able to show that injection of autologous adipose-tissue derived MSCs not only reduced joint pain and improved function but also led to cartilage regeneration [39]. MSCs therefore seem a promising target for cell-based approaches due to their regenerative but also immunomodulatory properties.

We have previously demonstrated elevated IL-6 secretion upon cocultivation of CD4+ T-cells enriched in regulatory T-cells with MSCs [14] as one possible mechanism for the stabilization of this population. We postulated that paracrine effects rather than cell-to-cell interactions may be dominant mechanism of interaction between T-cells and MSCs, which is supported by findings that conditioned medium from adipose-tissue derived MSCs can induce FoxP3+ Tregs [40]. IL-6 downregulation has also been shown to result in MSC immunoprivilege [41] and may additionally be an adaptive mechanism to MSC senescence [42]. The increased secretion of this cytokine observed in CD4+ T-cell cocultures may therefore be crucial to the regulatory properties of MSCs with regard to their HLA-DR expression and prior expansion.

Another cytokine that is associated with MSC immunomodulation is TGF-*β* [43]. A study by Svobodova et al. demonstrated that the MSC-induced generation of FoxP3+ Tregs from CD4+ T-cells was mediated by IL-6 and TGF-*β* [44]. TGF-*β* secretion however was only increased in BM-MSC/CD4+ cocultures upon stimulation in our study and varied importantly between the cultures. Given the fact that both SM-MSCs and BM-MSCs were able to recruit naïve Tregs from the CD4+ pool and no significant differences in TGF-*β* secretion of CD4+ T-cells and the respective MSC cocultures could be detected, this cytokine does not seem to play the same crucial role as IL-6 in this setting. It must however be stated that the high variation between the cultures may have hidden an effect.

## 5. Conclusions

Synovium derived MSCs from OA patients represent a distinct population regarding their surface marker distribution and immunomodulatory potential. Although bone-marrow and synovium derived MSCs show similarities in altering the cytokine profile of activated T-cells and in their potential to recruit naïve regulatory T-cells from the CD4+ pool in vitro, SM-MSCs exert antiproliferative effects upon T-cell activation. Also, compared to BM-MSCs, HLA-DR expression on SM-MSCs is markedly lower, suggesting potentially lower immunogenicity. In vivo, BM-MSCs and SM-MSCs thus may exert shared, but slightly different immunoregulatory properties. This makes SM-MSCs a promising target when pursuing cell-based approaches for modulating inflammatory responses.

## Supplementary Material

Antibodies and isotype antibodies used for flow cytometry.

## Figures and Tables

**(a):** 

Day 0	BM-MSCs	SM-MSCs	Wilcoxon BM-MSCs/SM-MSCs
CD14	4.87 ± 2.44	7.26 ± 5.31	*p* = 0.093
CD19	9.59 ± 8.67	11.79 ± 13.13	*p* = 0.8
CD34	1.04 ± 0.45	1.47 ± 0.57	**p** = 0.047
CD45	1.63 ± 0.61	1.91 ± 1.11	*p* = 0.44
CD73	99.84 ± 0.15	99.95 ± 0.05	**p** = 0.036
CD90	94.1 ± 4.3	98.01 ± 1.82	**p** = 0.022
CD105	99.86 ± 0.12	99.69 ± 0.19	**p** = 0.011
CD146	79.41 ± 7.49	15.95 ± 9.56	**p** = 0.005
HLA-ABC	99.94 ± 0.04	99.92 ± 0.08	*p* = 0.2
HLA-DR	52.38 ± 23.24	31.54 ± 23.13	**p** = 0.007
7-AAD	8.27 ± 5.93	3.6 ± 2.84	**p** = 0.017

**(b):** 

Day 5	BM-MSCs/CD4+	Control BM-MSCs	Wilcoxon BM-MSCs d5-d0/d5-control
CD14	1.02 ± 0.24	1.17 ± 1	**p** = 0.005/*p* = 0.51
CD19	34.15 ± 16.55	39.26 ± 20.81	**p** = 0.005/*p* = 0.17
CD45	2.32 ± 0.64	1.46 ± 0.79	*p* = 0.1/**p** = 0.007
CD73	99.19 ± 0.33	99.72 ± 0.17	**p** = 0.005/**p** = 0.005
CD90	88.43 ± 8.99	94.24 ± 5.09	**p** = 0.007/**p** = 0.005
CD105	99.65 ± 0.20	99.78 ± 0.14	**p** = 0.013/*p* = 0.05
CD146	39.97 ± 18.13	53.25 ± 19.58	**p** = 0.005/**p** = 0.005
HLA-ABC	98.56 ± 1.95	98.63 ± 2.20	**p** = 0.008/*p* = 0.11
HLA-DR	71.13 ± 16.16	66.13 ± 18.34	**p** = 0.005/**p** = 0.007
7-AAD	5.06 ± 3.41	11.15 ± 8.91	*p* = 0.074/**p** = 0.0069

**(c):** 

Day 5	SM-MSCs/CD4+	Control SM-MSCs	Wilcoxon SM-MSCs d5-d0/d5-control
CD14	0.79 ± 0.12	0.79 ± 0.17	**p** = 0.005/*p* = 0.48
CD19	15.04 ± 11.42	16.71 ± 11.98	*p* = 0.2/*p* = 0.17
CD34	1.42 ± 1.43	3.05 ± 2.53	*p* = 0.54/**p** = 0.005
CD45	1.9 ± 0.53	1.3 ± 0.21	*p* = 0.8/**p** = 0.005
CD73	99.39 ± 0.29	98.56 ± 4.06	**p** = 0.005/*p* = 0.007
CD90	96.38 ± 2.96	98.59 ± 1.09	*p* = 0.07/**p** = 0.005
CD105	99.38 ± 0.35	99.67 ± 0.15	**p** = 0.041/**p** = 0.005
CD146	1.08 ± 0.56	1.93 ± 0.91	**p** = 0.005/**p** = 0.005
HLA-ABC	99.16 ± 0.33	99.01 ± 1.56	**p** = 0.008/*p* = 0.139
HLA-DR	42.53 ± 24.45	35.11 ± 21.60	**p** = 0.005/**p** = 0.005
7-AAD	1.44 ± 0.63	2.89 ± 2.36	**p** = 0.037/**p** = 0.022
