# Supplementary material for: Mesenchymal Stromal Cells from Osteoarthritic Synovium Are a Distinct Population Compared to Their Bone-Marrow Counterparts regarding Surface Marker Distribution and Immunomodulation of Allogeneic CD4+ T-Cell Cultures
Source: Stem Cells Int. 2016 Jul 19;2016:6579463. doi: 10.1155/2016/6579463 (PMC4969547; doi:10.1155/2016/6579463)
Supplement: Supplementary file 1 — Antibodies and isotype antibodies used for flow cytometry. [file 6579463.f1.pdf]

Supplemental Table 1

Antibodies used for flow cytometry. AH: anti-human Ms: Mouse. ICAb: isotype control antibody.

Surface antigen	Color	Isotype	Company
**CD90**	FITC	AH Ms IgG1	BD Biosciences, Heidelberg, Germany
**CD73**	PE	AH Ms IgG1	BD Biosciences, Heidelberg, Germany
**CD105**	APC	AH Ms IgG1	BioLegend, San Diego, USA
**CD14**	FITC	AH Ms IgG2a	BD Biosciences, Heidelberg, Germany
**CD34**	PE	AH Ms IgG1	BD Biosciences, Heidelberg, Germany
**CD45**	PerCP	AH Ms IgG2a	Miltenyi Biotec, Bergisch Gladbach, Germany
**HLA-ABC**	Pacific Blue	AH Ms IgG2a	Biolegend, San Diego, USA
**CD146**	PE	AH Ms IgG1	BD Biosciences, Heidelberg, Germany
**CD19**	APC	AH Ms IgG1	Miltenyi Biotec, Bergisch Gladbach, Germany
**HLA-DR II**	APCCy7	AH Ms IgG2a,	Biolegend, San Diego, USA
**CD4**	FITC	AH Ms IgG2a	Miltenyi Biotec, Bergisch Gladbach, Germany
**Fox P3**	PE	AH Ms IgG1	BD Biosciences, Heidelberg, Germany
**CD127**	PeCy7	AH Ms IgG1	BioLegend San Diego, USA
**CD25**	APC	AH Ms IgG1	BD Biosciences, Heidelberg, Germany
**CD45 RO**	PE	AH Ms IgG2a	Biolegend San Diego, USA

**CD45 RA**	APCH7	AH Ms IgG2b	BD Biosciences, Heidelberg, Germany
**CD8**	VioBlue	AH Ms IgG2a	Miltenyi Biotec, Bergisch Gladbach, Germany
**CD14**	FITC	AH Ms IgG2a	BD Biosciences, Heidelberg, Germany
**CD19**	PE	AH Ms IgG1	Miltenyi Biotec, Bergisch Gladbach, Germany
**CD16**	PE-Cy7	AH Ms IgG1	BD Biosciences, Heidelberg, Germany
**CD56**	APC	AH Ms IgG1	Miltenyi Biotec, Bergisch Gladbach, Germany
**CD4**	APC-Cy7	AH Ms IgG1	BD Biosciences, Heidelberg, Germany
**IgG1 IcAb**	APC	MS IgG1	Miltenyi Biotec, Bergisch Gladbach, Germany
**IgG1 IcAb**	APC-Cy7	MS IgG1	BD Biosciences, Heidelberg, Germany
**IgG1 IcAb**	FITC	MS IgG1	BD Biosciences, Heidelberg, Germany
**IgG1 IcAb**	PE	MS IgG1	BD Biosciences, Heidelberg, Germany
**IgG1 IcAb**	PE-Cy7	MS IgG1	BD Biosciences, Heidelberg, Germany
**IgG2a IcAb**	APC-Cy7	MS IgG2a	BD Biosciences, Heidelberg, Germany
**IgG2a IcAb**	FITC	MS IgG2a	BD Biosciences, Heidelberg, Germany
**IgG2a IcAb**	FITC	MS IgG2a	Miltenyi Biotec, Bergisch Gladbach, Germany
**IgG2a IcAb**	PacificBlue	MS IgG2a	BioLegend, San Diego, CA, USA
**IgG2a IcAb**	PE	MS IgG2a	Miltenyi Biotec, Bergisch Gladbach, Germany
**IgG2a IcAb**	PerCP	MS IgG2a	Miltenyi Biotec, Bergisch Gladbach, Germany

**IgG2a IcAb**	VioBlue	MS IgG2a	Miltenyi Biotec, Bergisch Gladbach, Germany
**IgG2b IcAb**	APCH7	MS IgG2b	BD Biosciences, Heidelberg, Germany

Supplemental Table 2

Mean cytokine levels in stimulated vs. unstimulated cultures at day 5.

Results are displayed as mean cytokine concentration in pg/ml ±SD.

<TDL: Results beyond theoretical detection limit of the CBA assay.

	**CD4+ unstimulated**	**CD4+/ BM-MSC unstimulated**	**CD4+/ SM-MSC unstimulated**
IL-2	2.53± 1.23	5.89± 5.01	5.04±3.36
IL-4	1.12± 0.65 (<TDL)	0.23± 0.52 (<TDL)	0.26± 0.58(<TDL)
IL-6	4.41± 4.1	2644.52± 2522.8	509.81± 333.02
IL-10	2.36± 0.74 (<TDL)	2.01± 0.67 (<TDL)	2.49± 0.73 (<TDL)
IL-17a	3.18± 3.35 (<TDL)	0.76± 1.12 (<TDL)	1.59± 2.37 (<TDL)
TNFα	1.98± 0.86 (<TDL)	1.83± 0.31 (<TDL)	1.51± 0.16 (<TDL)
IFNγ	0.73± 1.05 (<TDL)	0.22± 0.48 (<TDL)	0.61± 0.87 (<TDL)
TGFβ	183.72± 73.22	302.73± 93.55	241.56± 80.19
	**CD4+ stimulated**	**CD4+/ BM-MSC stimulated**	**CD4+/ BM-MSC stimulated**
IL-2	8217.73± 4944.97	1573.95± 678.27	1863.89± 659.38
IL-4	29.19± 11.38	20.36± 5.15	20.8± 13.41
IL-6	131.28± 67.94	481503.5± 141513.5	248600.92± 136045.99
IL-10	238.84± 209.37	270.87± 207.46	183.86± 91.79
IL-17a	289.05± 204.44	989.54± 765.43	335.22± 187.96
TNFα	475.54± 300.78	72.03± 28.17	71.04± 30.86
IFNγ	1780.39± 1290.48	1054.12± 866.36	690.62± 413.63
TGFβ	194.8± 57.79	356.68± 119.12	271.68± 60.81
